# Morphological features of large layer V pyramidal neurons in cortical motor-related areas of macaque monkeys: analysis of basal dendrites

**DOI:** 10.1038/s41598-021-83680-5

**Published:** 2021-02-18

**Authors:** Yu Takata, Hiroshi Nakagawa, Taihei Ninomiya, Hajime Yamanaka, Masahiko Takada

**Affiliations:** 1 Systems Neuroscience Section, Primate Research Institute, Kyoto University, Inuyama, Aichi 484-8506 Japan; 2 Department of Molecular Neuroscience, World Premier International Immunology Frontier Research Center, Osaka University, 3-1 Yamadaoka, Suita, Osaka 565-0871 Japan; 3 Department of Developmental Physiology, National Institute for Physiological Sciences, Okazaki, 444-8585 Japan; 4 Department of Physiological Sciences, School of Life Science, The Graduate University for Advanced Studies (SOKENDAI), Hayama, Kanagawa 240-0193 Japan

**Keywords:** Neuroscience, Neurology

## Abstract

In primates, large layer V pyramidal neurons located in the frontal motor-related areas send a variety of motor commands to the spinal cord, giving rise to the corticospinal tract, for execution of skilled motor behavior. However, little is known about the morphological diversity of such pyramidal neurons among the areas. Here we show that the structure of basal dendrites of the large layer V pyramidal neurons in the dorsal premotor cortex (PMd) is different from those in the other areas, including the primary motor cortex, the supplementary motor area, and the ventral premotor cortex. In the PMd, not only the complexity (arborization) of basal dendrites, i.e., total dendritic length and branching number, was poorly developed, but also the density of dendritic spines was so low, as compared to the other motor-related areas. Regarding the distribution of the three dendritic spine types identified, we found that thin-type (more immature) spines were prominent in the PMd in comparison with stubby- and mushroom-type (more mature) spines, while both thin- and stubby-type spines were in the other areas. The differential morphological features of basal dendrites might reflect distinct patterns of motor information processing within the large layer V pyramidal neurons in individual motor-related areas.

## Introduction

Pyramidal neurons are the main projection neurons in the cerebral cortex. Thus, various lines of information processed in a given cortical area are conveyed to other cortical areas or subcortical regions through axonal branches of the pyramidal neurons. Transmission of such information from neuron to neuron takes place at synapses, and postsynaptic neurons receive it through their dendrites and dendritic spines.

Layer V is a major output layer of the cerebral cortex. In primates, large layer V pyramidal neurons in the motor-related areas of the frontal lobe, including the primary motor cortex (M1), the supplementary motor area (SMA), and the dorsal and ventral divisions of the premotor cortex (PMd, PMv), send their axons extensively to the brainstem and the spinal cord for control of voluntary movements^1–5^. These layer V pyramidal neurons have many dendritic spines, which are distributed more frequently on their basal than apical dendrites^6^. It has been reported in rodents that the basal dendrites of large layer V pyramidal neurons receive inputs from their neighboring neurons^7^ and layer II/III pyramidal neurons^8^. Such inputs through the basal dendrites may exert a strong impact on activity of the layer V pyramidal neurons. To reveal the structural basis for control of large layer V pyramidal neuron activity, it is essential to define the morphology of their basal dendrites and dendritic spines.

In general, wide variations in pyramidal neuron structure depend on the areal and laminar specificity of the cortex. For example, the morphological features of basal dendrites of pyramidal neurons in layer III, i.e., their complexity and spine number, vary among the visual cortical areas of primates^9^, thus reflecting a certain functional diversity of individual areas. Likewise, the frontal motor-related areas are involved in different aspects of motor control^10–13^. In addition, each area receives somewhat distinct inputs from other cortical areas^14,15^. These facts lead us to hypothesize that the structure of large pyramidal neurons in layer V may be different among the areas. However, little is known about the differential morphological characteristics of large layer V pyramidal neurons in the motor-related areas. To understand how the morphological features of these pyramidal neurons represent the functional role(s) specific to each area, we investigated the possible differences in pyramidal neuron structure, with particular reference to the basal dendrites, among the M1, SMA, PMd, and PMv of macaque monkeys. In the present study, the morphology of basal dendrites of the large layer V pyramidal neurons was analyzed and compared in individual motor-related areas, especially in their digit regions.

## Results

Before analyzing the morphological features of large layer V pyramidal neurons giving rise to the corticospinal tract (CST), especially for digit movement, we performed two preparatory experiments: retrograde labeling of CST neurons and intracortical microstimulation (ICMS) mapping in the motor-related areas of the frontal lobe.

### Retrograde labeling of CST neurons

**Figure 1:**
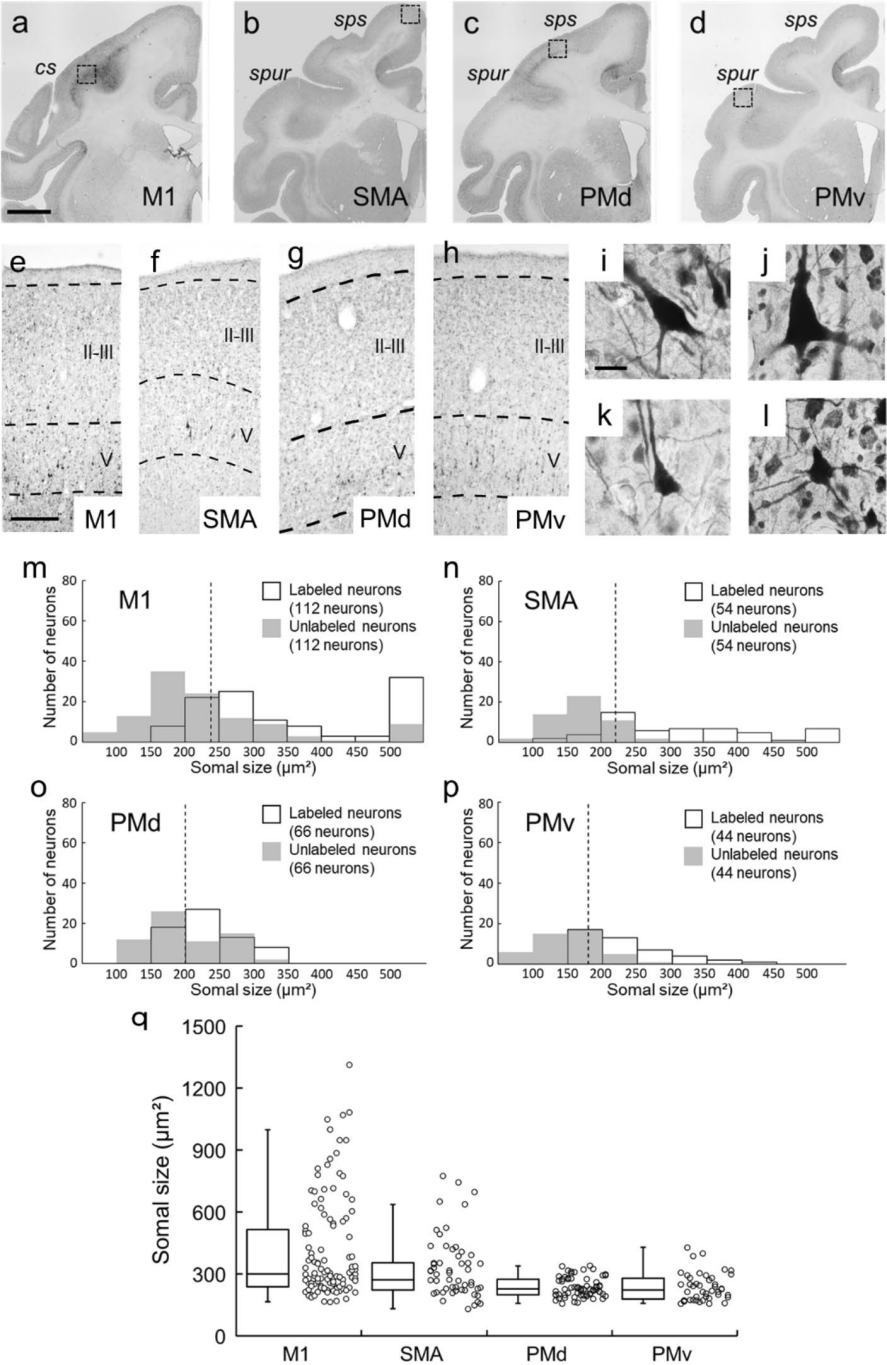
Retrograde labeling of CST neurons in frontal motor-related areas and their somal size measurement. (**a**–**d**) Low-magnification photos of retrograde labeling of CST neurons in the frontal motor-related areas after rabies injections into the C6–T1 levels of the spinal cord. (**a**) M1, (**b**) SMA, (**c**) PMd, (**d**) PMv. Scale bar, 5 mm. cs, central sulcus; sps, superior precentral sulcus; spur, spur of the arcuate sulcus. (**e**–**h**) Higher-magnification photos in the M1 (**e**), SMA (**f**), PMd (**g**), and PMv (**h**). Scale bar, 500 µm. II, layer II; III, layer III; V, layer V. (**i**–**l**) Representative CST neurons (i.e., large layer V pyramidal neurons) taken respectively from dotted squares of panels (**a**–**d**). Scale bar, 20 µm. (**m**–**p**) Measurement of the somal size of labeled CST neurons in the motor-related areas. As control, the same number of unlabeled layer V pyramidal neurons was sampled from each area. Data were obtained in 112, 54, 66, and 44 neurons sampled from 14 sections through the M1 (**m**), SMA (**n**), PMd (**o**), and PMv (**p**), respectively. Dotted line indicates the first quartile for the labeled CST neurons in each area. (**q**) Distribution of the somal size of labeled CST neurons in each area (left, box plot; right, dot plot).

In the present study, it is critical to sample CST neurons out of pyramidal neurons in layer V of the frontal motor-related areas. To solve this issue, we employed retrograde transport of rabies virus to examine the largeness of CST neurons in individual motor-related areas projecting to the cervical enlargement. The use of rabies virus for labeling CST neurons was meritorious in that this virus is taken up specifically from axon terminals, but not from passing fibers, and provides the explicit Golgi-like morphology of labeled neurons with the somal size unchanged^16–18^. After rabies injections into the cervical enlargement, especially into the C6–T1 levels for digit innervation, retrogradely labeled CST neurons were observed in the motor-related areas (Fig. 1a–l). All labeled neurons were confined to layer V across the areas, indicating that only monosynaptically-connected neurons were traced in this monkey. We sampled 112, 54, 66, and 44 neurons from the M1, SMA, PMd, and PMv, respectively, and measured their somal size using Neurolucida explorer. The same number of unlabeled neurons was sampled from layer V of each area. As shown in Fig. 1m–p, the somal size of the labeled CST neurons was 416.54 ± 23.42 μm^2^ for the M1, 331.78 ± 20.67 μm^2^ for the SMA, 235.55 ± 5.81 μm^2^ for the PMd, and 236.69 ± 10.13 μm^2^ for the PMv (for the somal size distribution, see also Fig. 1q), while that of the unlabeled neurons was 268.05 ± 21.55 μm^2^, 175.4 ± 7.64 μm^2^, 203.06 ± 6.47 μm^2^, and 154.47 ± 6.70 μm^2^ for individual areas, respectively. The somal size of the labeled CST neurons was significantly larger than that of the unlabeled neurons in each motor-related area (TukeyHSD; *p* < 0.01). Based on these data, putative CST neurons, the somal size of which was larger than the first quartile for the labeled CST neurons (238.29 μm^2^, 221.49 μm^2^, 199.78 μm^2^, and 179.6 μm^2^ for individual areas), were selected for their morphological analyses.

### ICMS mapping

**Figure 2:**
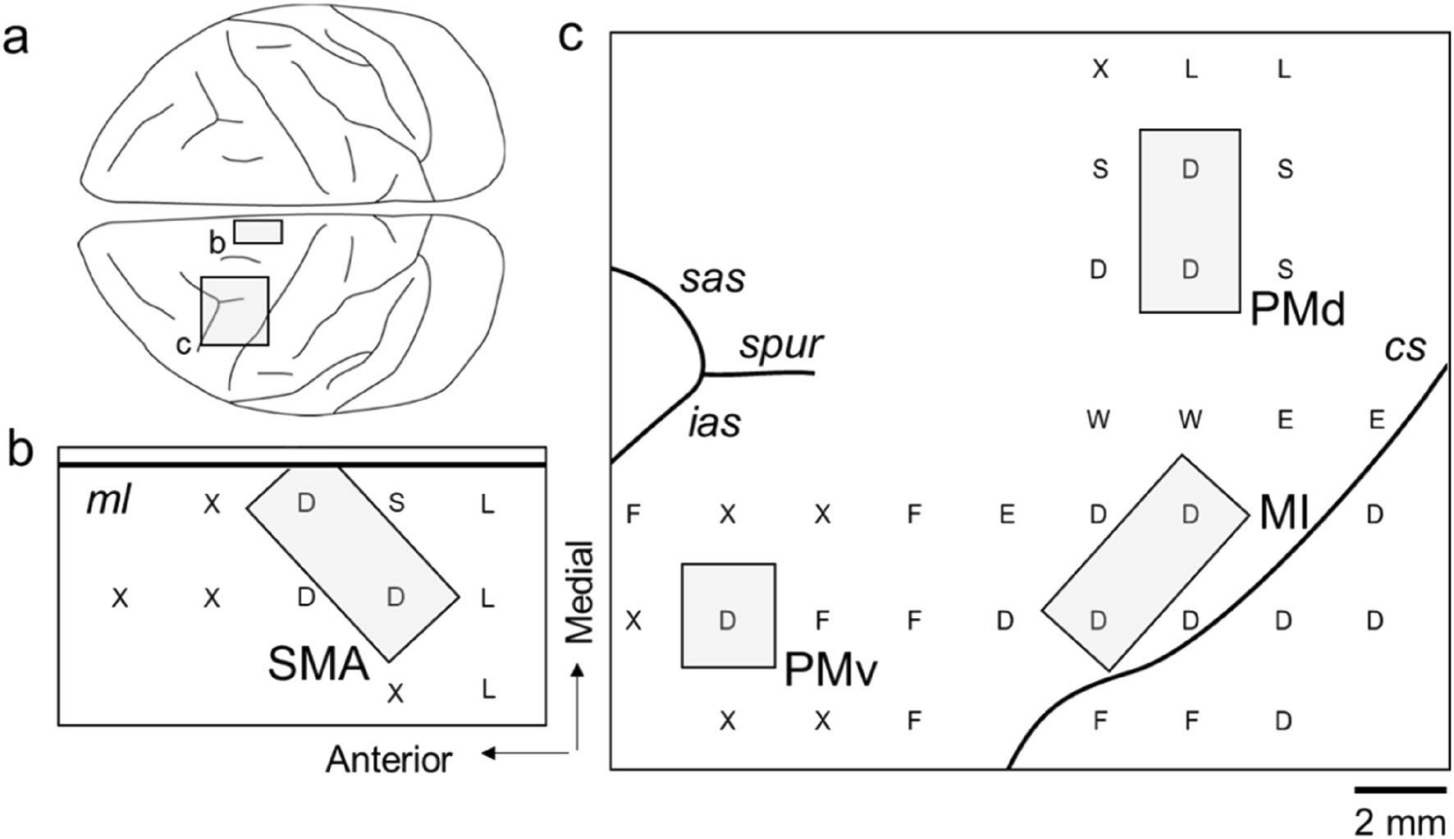
ICMS mapping of frontal motor-related areas. (**a**) Dorsal view of the monkey brain showing the frontal regions examined for ICMS mapping. (**b**) Results of ICMS mapping for identification of the digit representation of the SMA. (**c**) Results of ICMS mapping for identification of digit representations of the M1, PMd, and PMv. The identified digit regions (in gray) are taken for morphological analyses of the large layer V pyramidal neurons. The body parts of which movements were evoked by ICMS are indicated as follows: D, digits; E, elbow; F, face; L, leg; S, shoulder; W, wrist; X, no response. ias, inferior limb of the arcuate sulcus; ml, midline; sas, superior limb of the arcuate sulcus. Other abbreviations are as in Fig. 1.

In the present study, it is prerequisite to dissociate the digit region in each of the motor-related areas as accurately as possible. To achieve this purpose, we carried out ICMS to identify the digit regions in individual motor-related areas. In a representative case shown in Fig. 2, movements of the digits were evoked from five loci within the M1, three loci within the SMA, three loci within the PMd, and one locus within the PMv. According to the results of ICMS mapping, we determined the border between the digit region and other body-part regions in each area and dissected out a tissue block containing its digit representation alone for morphological analyses of putative CST neurons (Fig. 2).

### Complexity of basal dendrites

**Figure 3:**
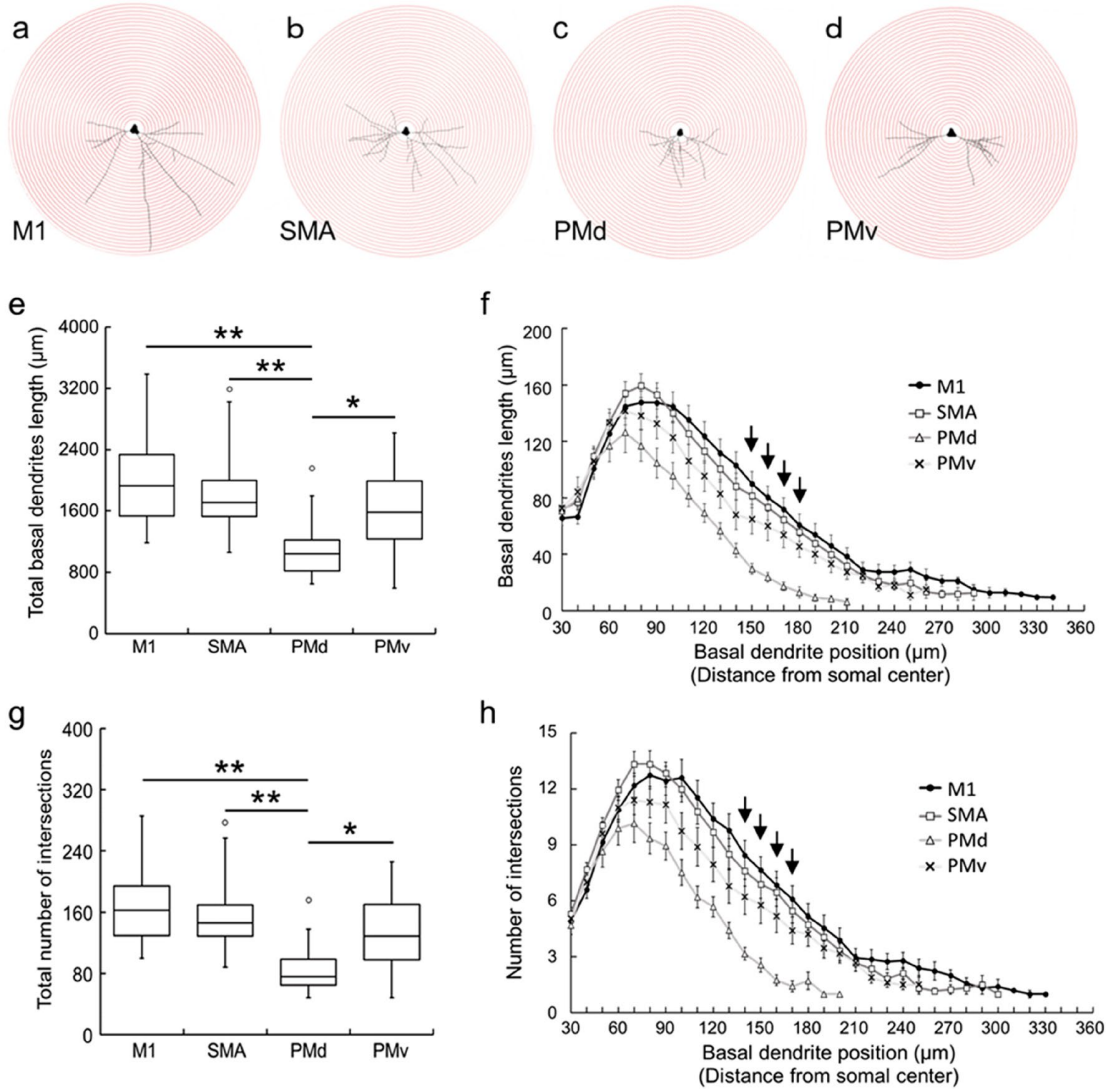
Complexity of basal dendrites of large layer V pyramidal neurons in frontal motor-related areas. (**a**–**d**) Plots of Sholl analysis of basal dendrites of large layer V pyramidal neurons. (**a**) M1, (**b**) SMA, (**c**) PMd, (**d**) PMv. For Sholl analysis, concentric circles were utilized starting at 30 µm away from the center of soma and increasing radii by 10 µm. (**e**) Total length of basal dendrites in the motor-related areas. Taken from 20 neurons in each area. Tukey–Kramer method. *p < 0.05, **p < 0.01. (**f**) Basal dendrites length at every 10-µm position. Expressed as the summation of the length of single basal dendrites measured per 10 µm. Error bars denote SEM. Arrows indicate the positions where the basal dendrites length is significantly shorter in the PMd than in the other three motor-related areas (post hoc pairwise Tukey–Kramer method comparison of the basal dendrites length; *p* < 0.05). At most of the other positions, the basal dendrites length is significantly lower in the PMd than in at least one motor-related area. (**g**) Total number of intersections of basal dendrites in the motor-related areas. Taken from 20 neurons in each area. Tukey–Kramer method. *p < 0.05, **p < 0.01. (**h**) Intersection number at every 10-µm position. Expressed as the summation of the number of intersections on the concentric circles. Error bars denote SEM. Arrows indicate the positions where the intersection number is significantly smaller in the PMd than in the other three motor-related areas (post hoc pairwise Tukey–Kramer method comparison of the intersection number; *p* < 0.05). At most of the other positions, the intersection number is significantly lower in the PMd than in at least one motor-related area.

**Figure 4:**
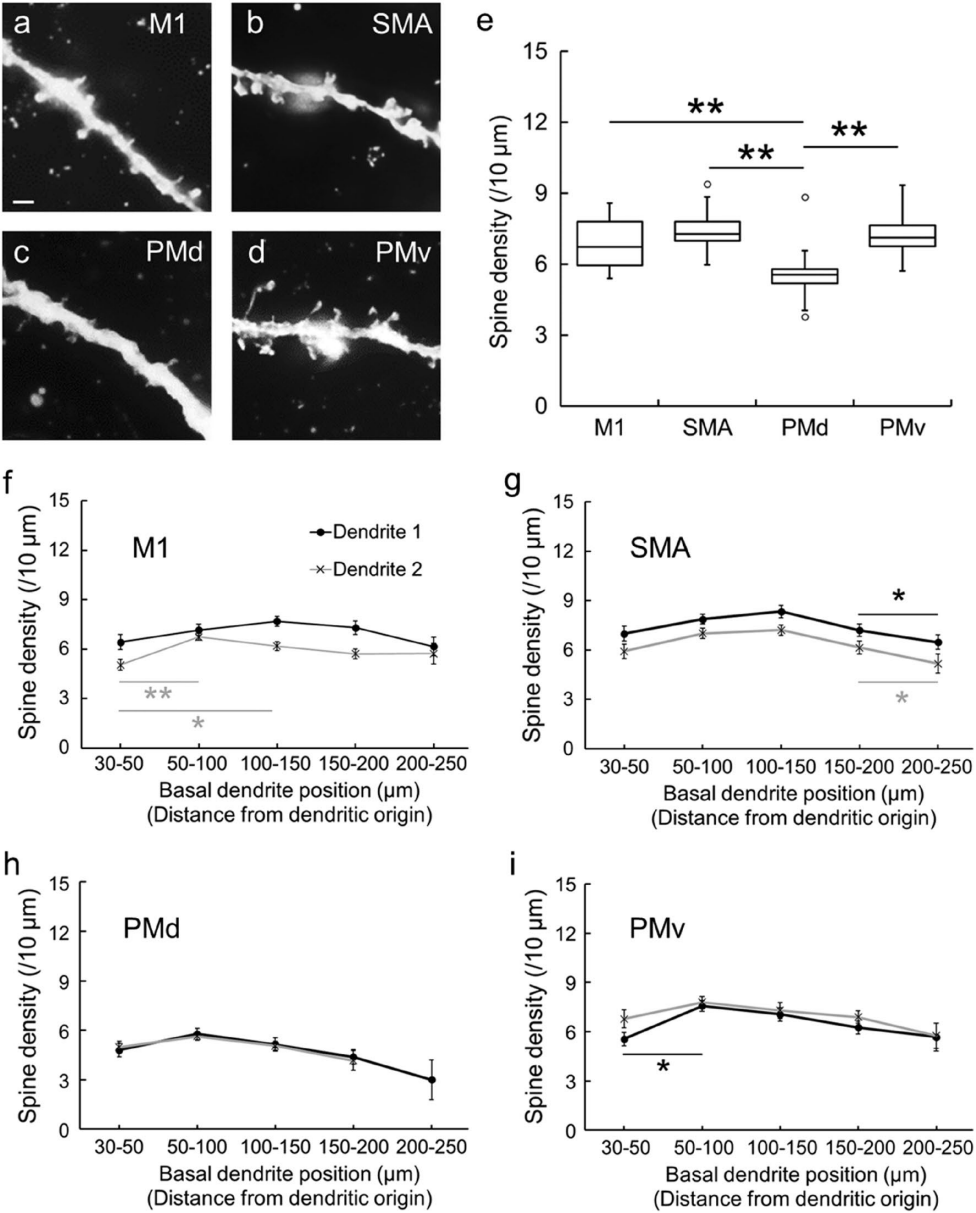
Spine density of basal dendrites of large layer V pyramidal neurons in frontal motor-related areas. (**a**–**d**) Representative morphology of basal dendrite spines of Golgi-impregnated large layer V pyramidal neurons in the M1 (**a**), SMA (**b**), PMd (**c**), and PMv (**d**). Scale bar, 2 µm. (**e**) Spine density of basal dendrites in the motor-related areas. Data on two dendrites taken from each of 20 neurons in individual areas. Expressed as the mean number of spines per 10-µm segment of single dendrites. Tukey–Kramer method. **p < 0.01. (**f**–**i**) Spine density of two basal dendrites on every 20- or 50-µm segment. Taken from 20 neurons in the M1 (**f**), SMA (**g**), PMd (**h**), and PMv (**i**). Error bars denote SEM. *p < 0.05, **p < 0.01.

We selected 20 putative CST neurons from each motor-related area based on the somal size: 464.75 ± 30.22 μm^2^ for the M1; 460.89 ± 37.68 μm^2^ for the SMA; 287.74 ± 8.02 μm^2^ for the PMd; and 319.47 ± 15.76 μm^2^ for the PMv. We traced the basal dendrites of putative CST neurons within the digit regions of individual motor-related areas, as the full length of each dendrite appeared to be followed successfully in single sections. We then surveyed the complexity of basal dendrites, i.e., total dendritic length and intersection number, by means of Sholl analysis (for details, see Methods; Fig. 3a–d). Data obtained were as follows: (1) The total length of basal dendrites in the PMd was significantly shorter than in the M1, SMA, and PMv (TukeyHSD; *p* < 0.05; Fig. 3e). There were no significant differences in the total basal dendrites length among the M1, SMA, and PMv (TukeyHSD; Fig. 3e); (2) The basal dendrites length around 150–180 µm apart from the somal center was significantly shorter in the PMd than in the other motor-related areas (TukeyHSD; *p* < 0.05; Fig. 3f); (3) The total number of intersections in the PMd was significantly smaller than in the M1, SMA, and PMv (TukeyHSD; *p* < 0.05; Fig. 3g). There were no significant differences in the total intersection number among the M1, SMA, and PMv (TukeyHSD; Fig. 3g); (4) The intersection number around 140–170 µm apart from the somal center was significantly smaller in the PMd than in the other motor-related areas (TukeyHSD; *p* < 0.05; Fig. 3h); and (5) In close proximity of the soma (~ 30 µm from the somal center), there were no significant differences in the intersection number among the motor related areas (TukeyHSD; Fig. 3h).

### Density of dendritic spines

**Figure 5:**
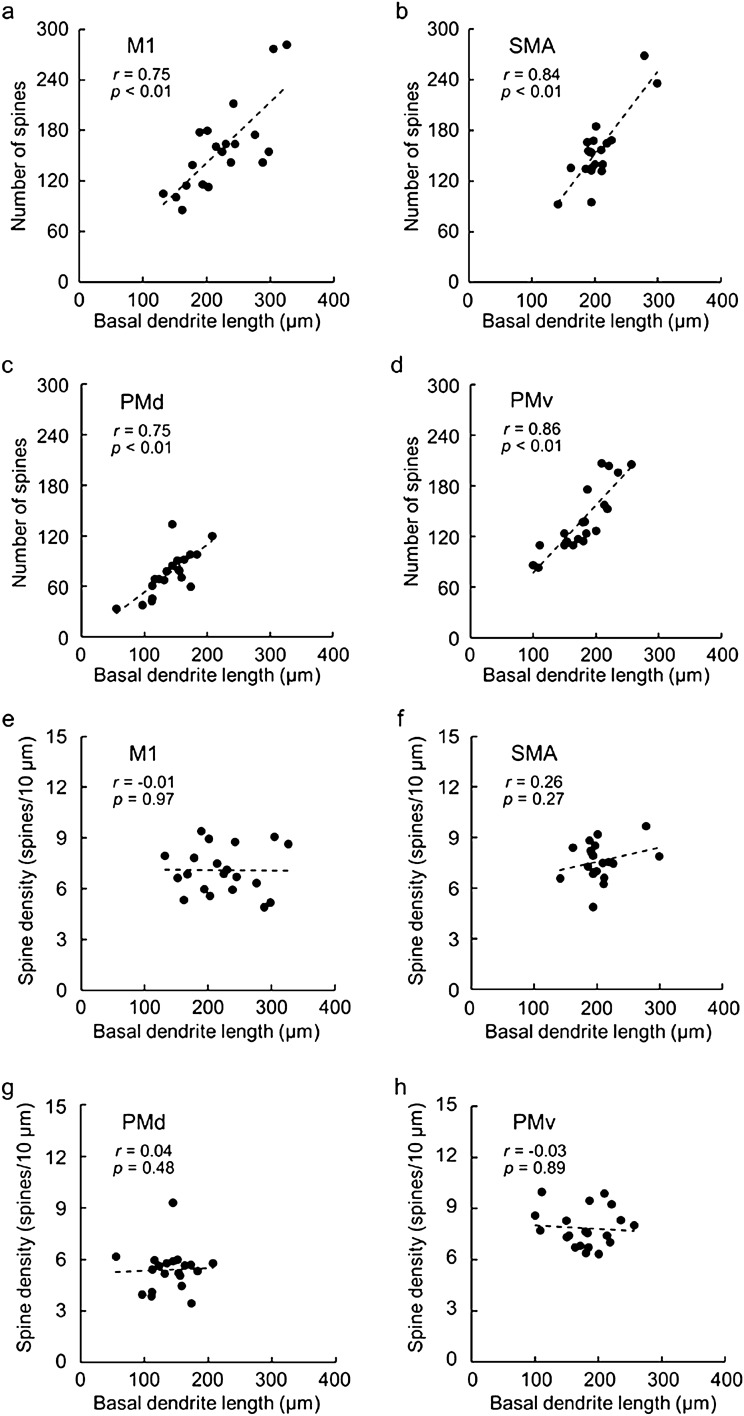
Correlations between basal dendrite length and spine number/density of large layer V pyramidal neurons. (**a**–**d**) Correlations between the basal dendrite length and the spine number in the M1 (**a**), SMA (**b**), PMd (**c**), and PMv (**d**). Taken from 20 neurons of one dendrite (Dendrite 1 in Fig. 4f–i) in each area. (**e**–**h**) Correlations between the basal dendrite length and the spine density in M1 (**e**), SMA (**f**), PMd (**g**), and PMv (**h**). Taken from 20 neurons of one dendrite (Dendrite 1 in Fig. 4f–i) in each area.

The density of dendritic spines was analyzed for the basal dendrites of CST neurons within the digit regions of individual motor-related areas using Neurolucida explorer (Fig. 4a–d). In the present experiments, we counted the number of spines on two dendrites in each area and converted it to the value per 10-µm segment. It was found that the density of dendritic spines in the PMd was significantly lower than in the other motor-related areas (TukeyHSD; *p* < 0.01; Fig. 4e). To confirm whether such a distribution pattern of dendritic spines might depend on the basal dendrite position, we examined the density of spines on every 20- or 50-µm segment. In each motor-related area, there was no significant difference in the spine density between the two dendrites (data not shown). Depending on the basal dendrite position, on the other hand, there were some differences in the spine density in the M1, SMA, and PMv (TukeyHSD; *p* < 0.05; Fig. 4f–i). Moreover, we analyzed the correlation between the spine number/density and the dendrite length for single basal dendrites of putative CST neurons. A strong positive correlation between the spine number and the dendrite length was detected in each of the motor-related areas (Pearson correlation coefficient; Fig. 5a–d). By contrast, no clear correlation between the spine density and the dendrite length was found in any area (Pearson correlation coefficient; Fig. 5e–h).

### Distribution of dendritic spine types in motor-related areas

**Figure 6:**
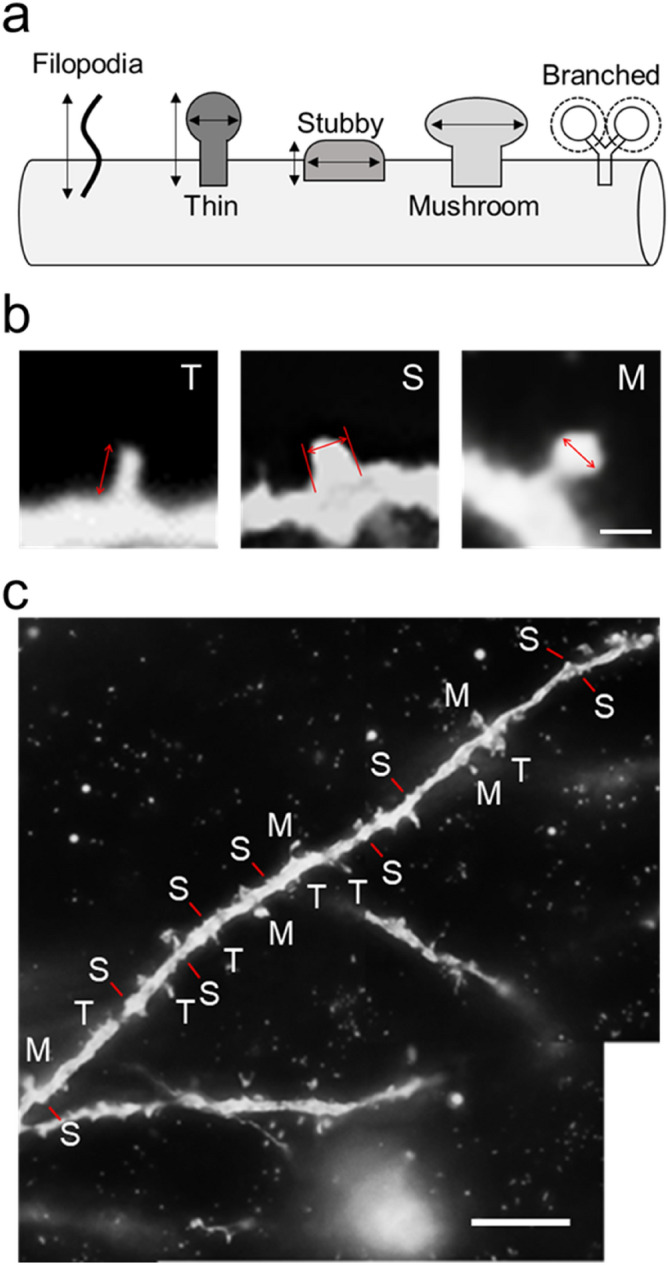
Five types of dendritic spines. (**a**) Schematic drawings of five spine types. (**b**) Typical examples of thin (T)-, stubby (S)-, and mushroom (M)-type spines. Arrows represent the length of a thin-type spine, and the width of a stubby- and a mushroom-type spine. Scale bar, 1 µm. (**c**) Thin (T)-, stubby (S)-, and mushroom (M)-type spines distributed on a single basal dendrite. Taken from large layer V pyramidal neuron in the M1. Scale bar, 10 µm. For the precise criteria on spine type classification, see the Methods.

**Figure 7:**
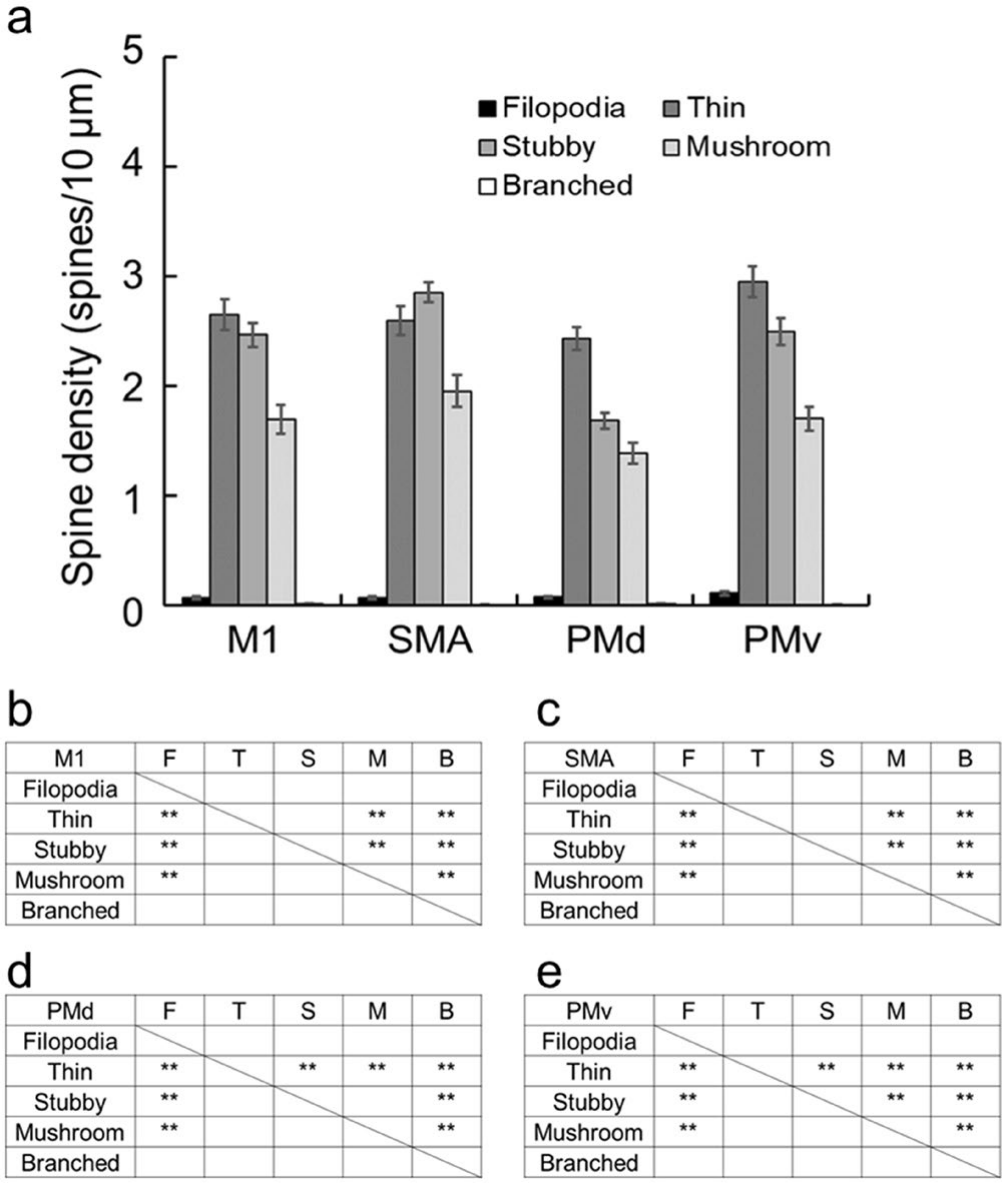
Distribution of dendritic spine types. (**a**) Density of five spine types in the basal dendrites of large layer V pyramidal neurons in the motor-related areas. Data on two dendrites taken from each of 20 neurons in individual areas. Expressed as the mean number of spines per 10-µm segment of single dendrites. Error bars denote SEM. (**b**–**e**) Cross tables showing the results of post hoc pairwise Tukey–Kramer method comparison of the distribution of the five spine types (F, filopodia; T, thin; S, stubby; M, mushroom; B, branched) in the M1 (**b**), SMA (**c**), PMd (**d**), and PMv (**e**). In these cross tables, asterisks indicate that the value for one spine type on rows is significantly higher than for other spine type(s) on columns. *p < 0.05, **p < 0.01. Note that both the filopodia and the branched types are quite a few or almost none in each of the motor-related areas, and that in the M1, SMA, and PMv, the density of thin- and stubby-type spines is comparable to each other and much higher than that of mushroom-type spines, but that in the PMd, the density of thin-type spines is significantly higher than those of stubby- and mushroom-type spines.

**Figure 8:**
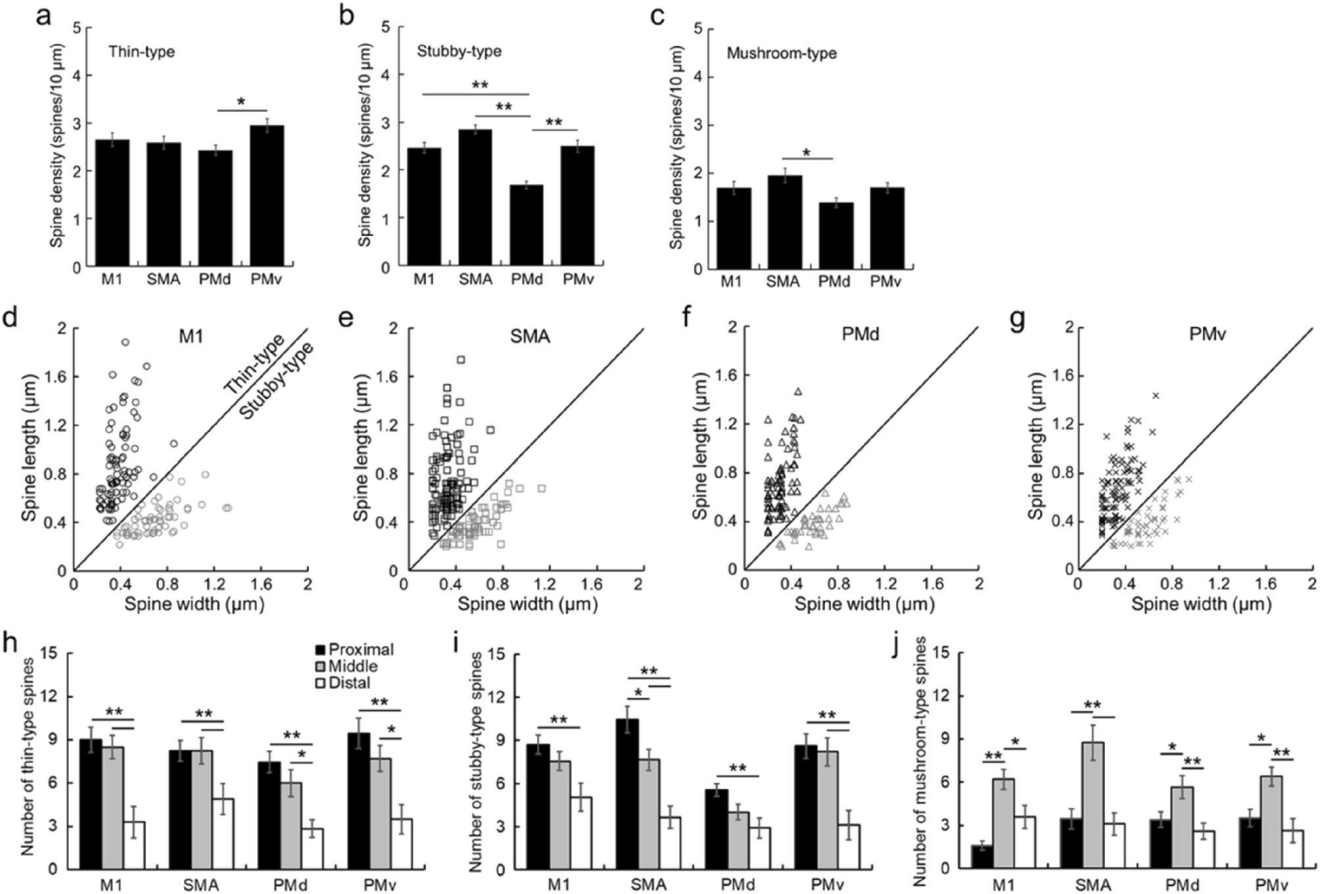
Density comparison of thin-, stubby-, and mushroom-type spines among frontal motor-related areas. (**a**–**c**) Density of thin-type (**a**), stubby-type (**b**), and mushroom-type (**c**) spines in the basal dendrites of large layer V pyramidal neurons in the motor-related areas. Expressed as in Fig. 7a. Error bars denote SEM. Tukey–Kramer method. *p < 0.05, **p < 0.01. Note that the density of stubby-type spines is much lower in the PMd than in the other areas. (**d**–**g**) Scatter plots of thin- and stubby-type spine morphology (length vs. width) for the M1 (**d**), SMA (**e**), PMd (**f**), and PMv (**g**). (**h**–**j**) Number of thin-type (**h**), stubby-type (**i**), and mushroom-type (**j**) spines on the proximal, middle, and distal segments of a single basal dendrite. Error bars denote SEM. *p < 0.05, **p < 0.01.

Finally, the distribution of the five dendritic spine types, i.e., the filopodia, thin, stubby, mushroom, and branched types (Fig. 6), was investigated in the motor-related areas. For each type, the spine density was expressed as the number per 10-µm segment of single basal dendrites. Both the filopodia and the branched types were only a few or almost none in each of the motor-related areas (TukeyHSD; *p* < 0.01; Fig. 7). The other three types of spines were consistently observed in all motor-related areas (Figs. 7a and 8). In the M1 and SMA, the density of thin- and stubby-type spines was comparable to each other, and these types of spines were much more abundant than mushroom-type spines (TukeyHSD; *p* < 0.01; Fig. 7a–c). On the other hand, the patterns of spine type distribution in the PMd and PMv were somewhat different from those in the M1 and SMA. The density of each of thin-, stubby-, and mushroom-type spines was relatively low in the PMd as compared to the other areas (Figs. 7a and 8). Particularly, the density of stubby-type spines was far lower in the PMd than in all of the M1, SMA, and PMv (TukeyHSD; *p* < 0.01; Fig. 8b). Within the PMd and PMv, the density of thin-type spines was significantly higher than those of stubby- and mushroom-type spines (TukeyHSD; *p* < 0.01; Fig. 7a,d,e). Also, there was no significant difference in the PMd between the density of stubby- and mushroom-type spines (Fig. 7a,d).

Regarding the spine morphology, both the length and the width of thin-type spines were significantly larger in the M1 than in the other motor-related areas (TukeyHSD; *p* < 0.01; Fig. 8d–g). Also, there were no significant differences in the morphology of thin-type spines among the SMA, PMd, and PMv (Fig. 8d–g). On the other hand, the width of stubby-type spines was significantly larger in the M1 than in the other areas (TukeyHSD; *p* < 0.05; Fig. 8d–g). With respect to the correlation between the distribution of spine types and the distance from the dendritic origin, the number of thin- and stubby-type spines at the distal segment was much smaller in all motor-related areas (TukeyHSD; *p* < 0.05; Fig. 8h–j). On the other hand, the number of mushroom-type spines at the middle segment was significantly larger in all areas (TukeyHSD; *p* < 0.05; Fig. 8h–j).

## Discussion

In the present study, we morphologically analyzed the large layer V pyramidal neurons by quantitatively comparing the structure of their basal dendrites, i.e., dendritic arbors and spines, in the motor-related areas of macaque monkeys. We selected representative neurons for analysis as accurately as possible by measuring the somal area of CST neurons retrogradely labeled from the C6–Th1 segments of the spinal cord and by identifying the digit region of each motor-related area with ICMS mapping. We have found that the complexity (arborization) of basal dendrites, i.e., the total dendritic length and intersection number, in the large layer V pyramidal neurons seems poorly developed in the PMd as compared to the other motor-related areas, including the M1, SMA, and PMv. Interestingly, it has been reported that the dendritic arborization of layer III pyramidal neurons in the PMd is more complex than in the M1^19^. These data suggest that the dendritic arborization of pyramidal neurons differs in a layer-dependent manner. We have further demonstrated that the spine density of basal dendrites in the large layer V pyramidal neurons is lower in the PMd than in the other motor-related areas. By contrast, it has been shown that the number of dendritic spines of layer III pyramidal neurons is larger in the PMd than in the M1^19^. A similar layer-specific diversity has also been observed in the cortical areas of macaque monkeys^20^. In our statistical analysis at the single dendrite level, we could detect a strong positive correlation between the dendritic length and the spine number of basal dendrites in each of the motor-related areas. However, no positive correlation between the dendritic length and the spine density was found in any area. This indicates that the density of dendritic spines does not depend on the dendritic length.

Manual dexterity, represented when manipulating a small object, is most developed in higher primates, including monkeys and humans. Accumulated evidence using monkeys implies that skilled motor behavior with the digits is achieved by neuronal activity in the frontal motor-related areas^21–25^. Functional imaging studies in humans have further reported that these motor-related areas are co-activated during fine digit movements^26,27^. However, it has been shown that the number of CST neurons projecting to the cervical enlargement is smaller in the PMd and PMv than in the M1 and SMA^28^. In favor of this, Morecraft et al. have demonstrated that CST terminals from the PMd and PMv are less dense in the cervical enlargement, compared with the M1 and SMA^3,4^. Thus, the PMd as well as the PMv might make a smaller contribution to dexterous movements of the digits.

In addition, we classified dendritic spines into five types according to the prior studies^29,30^ to explore the distribution pattern of these spine types on the basal dendrites. It should be noted here, however, that the present spine type classification was not performed three dimensionally by high-resolution fluorescent microscopy or electron microscopy, but was carried out under a two-dimensional light microscope. A recent work^31^ has described that there are two limitations in spine type classification using light microscopy. The first limitation is the resolution level, because the xy-plane resolution of a light microscope is limited up to 200 nm and the z-axis resolution is much lower. This may make it quite difficult to determine the spine size, especially filopodia- and thin-type spines. The second limitation is the direction of observation, because observation from the only one direction can lead to incorrect classification of spine types. Therefore, the same weakness is inherent in our analysis of the spine morphology. It has been well documented that the filopodia and thin types of spines contribute to the learning process, while the stubby, mushroom, and branched types of spines are involved in the memory formation^32,33^. Moreover, the mushroom-type spines have been implicated in long-term memory because they are more mature and stable. These overall results indicate that filopodia- and thin-type spines are more immature, whereas mushroom- and branched-type spines are more mature. In our analysis, all motor-related areas were commonly devoid of the filopodia and branched types, and conversely, rich in the thin, stubby, and mushroom types. We have further found that the density of thin-type spines is higher in the PMd compared with stubby- and mushroom-type spines, and that not only thin-type spines, but also stubby-type spines exhibit higher density than mushroom-type spines in the other motor-related areas. The present data suggest that the large layer V pyramidal neurons in the PMd may have a higher neuroplastic capability.

It has been shown in mice that the basal dendrites of large layer V pyramidal neurons receive presumably synchronized inputs from their neighboring neurons^7^, and diverse inputs from other cortical areas via layer II/III pyramidal neurons^8^. According to a previous electrophysiological work on motor learning, recurrent inputs to layer V pyramidal neurons could play an important role in their synchronized activity^34^. Therefore, the morphological diversity of basal dendrites and their spines might reflect distinct patterns of motor information processing within the large layer V pyramidal neurons, i.e., CST neurons, in the frontal motor-related areas and, also, a variety of motor commands to be issued from the CST neurons. In fact, previous studies have demonstrated that the motor-related areas other than the M1 modulate outputs of the M1 through interareal connections^35,36^, and that these areas make relatively small contributions to direct innervation over the cervical enlargement based on the number of CST neurons and the density of CST terminals^3,4^. However, the relationship between the structure and the function of basal dendrites of CST neurons is poorly understood. Of particular interest is that dendrites and dendritic spines of monkey cortical neurons have the capacity to change their morphology, number, density, and motility not only during development, but also in adulthood^37^. Moreover, it has been reported that the plastic change of dendritic spine morphology of mouse CST neurons occurs during motor recovery from spinal cord injury^38^. Thus, in-depth studies are needed to understand the correlation between CST neuron-related neuroplastic events and motor outputs.

## Methods

### Animals

Three adult macaque monkeys, one rhesus monkey (*Macaca mulatta*; male, 6.0 kg) and two Japanese monkeys (*Macaca fuscata*; one male, 7.0 kg; one female, 6.0 kg), were used for this study. The rhesus monkey was to specify the large layer V pyramidal neurons by evaluating the largeness of CST neurons arising from the frontal motor-related areas, and the Japanese monkeys were to analyze the morphology of basal dendrites of the large layer V pyramidal neurons. The experimental protocols were approved by the Animal Welfare and Animal Care Committee of Primate Research Institute, Kyoto University, and all experiments were conducted in accordance with the Guidelines for the Care and Use of Laboratory Primate (Ver. 3, 2010) set by the Primate Research Institute of Kyoto University, Japan.

### Retrograde labeling of CST neurons

To label retrogradely CST neurons in individual motor-related areas, the challenge-virus-standard (CVS)-11 strain of rabies virus was injected unilaterally into the cervical enlargement in a rhesus monkey. The virus was originally derived from the Center for Disease Control and Prevention (Atlanta, GA, USA) and was donated by Dr. Satoshi Inoue (The National Institute of Infectious Diseases, Tokyo, Japan). It has been demonstrated that the rabies strain CVS-11 is transsynaptically transported in the retrograde direction^16,18^. When the rate of retrograde transport for the viral batch used in the present study was calibrated by evaluating transneuronal labeling in the cortico-basal ganglia loop circuit in our previous work^39^, we concluded that the two-day survival period was appropriate to label monosynaptically-connected neurons. The titer of a viral suspension was 1.4 × 10^8^ focus-forming units (FFU)/ml. The monkey was sedated with a combination of ketamine hydrochloride (10 mg/kg, i.m.) and xylazine hydrochloride (1 mg/kg, i.m.), and then anesthetized with sodium pentobarbital (20 mg/kg, i.v.). Under aseptic conditions, the spinal cord between the C4 and the Th2 segment was exposed by laminectomy with the monkey fixed in a stereotaxic frame. By using a 10-μl Hamilton microsyringe, a total of eight penetrations were made just medial to the lateral funiculus of the C6–Th1 segments. In each penetration, a 0.5-μl viral suspension was infused at the depth of 4 mm and then 2 mm from the dorsal surface, and the microsyringe was kept in place for a few min. After the rabies injections, the back muscles and skin were sutured.

After a survival of two days, the monkey was anesthetized deeply with an overdose of sodium pentobarbital (50 mg/kg, i.v.) and perfused transcardially with 0.1 M phosphate-buffered saline (PBS; pH 7.4), followed by 10% formalin in 0.1 M phosphate buffer (pH 7.4). The brain was removed from the skull, post-fixed in the same fresh fixative overnight at 4 °C, and then saturated with 30% sucrose at 4 °C. The histochemical procedures for rabies visualization were as described elsewhere^40^. Briefly, the cerebral hemisphere contralateral to the rabies injections was serially cut into 60-μm-thick coronal sections on a freezing microtome. Every sixth section was first immersed in 0.3% H_2_O_2_ for 30 min. After several washes in PBS, the sections were immersed in 1% skim milk for 1 h and incubated overnight at 4 °C with rabbit anti-rabies virus antibody^41^ (diluted at 1:10,000) in PBS containing 0.1% Triton X-100 and 1% normal goat serum. The sections were then incubated for 2 h in the same fresh medium containing biotinylated goat anti-rabbit IgG antibody (diluted at 1:200; Vector Laboratories, Burlingame, CA, USA) and treated with the ABC Elite kit (Vector Laboratories, Burlingame, CA, USA) for 1.5 h. The sections were reacted in 0.05 M Tris-HCl buffer containing 0.04% 3,3′-diaminobenzidine, 0.04% nickel chloride, and 0.002% hydrogen peroxide to visualize rabies-labeled CST neurons. Finally, these sections were counterstained with 0.1% Neutral red. The adjacent series of the sections were Nissl-stained with 1% Cresyl violet to determine the areal and laminar boundaries of the motor-related areas.

### Measurement of somal area of CST neurons

The somal area of rabies-labeled CST neurons were measured in the M1, SMA, PMd, and PMv of the hemisphere opposite to the tracer injections. In 14 coronal sections, images of the CST neurons were traced and analyzed by using Neurolucida and Neurolucida explorer (MBF Bioscience, Williston, VT, USA). For the present measurement, a total of 112, 54, 66, and 44 labeled neurons were sampled from the M1, SMA, PMd, and PMv, respectively, and their somal size was measured with Neurolucida explorer. The same number of unlabeled neurons were sampled from layer V of each area. Somata of the CST neurons were circumscribed as previously reported^42^. All images were acquired with microscopes (for lower-power images, Biorevo BZ-9000, Keyence, Japan; for higher-power images, Axio Imager Z1, Carl Zeiss, Germany).

### ICMS mapping

In two Japanese monkeys, ICMS was performed to identify the digit regions of the motor-related areas electrophysiologically, as previously described^43^. Briefly, after sedation with ketamine hydrochloride (10 mg/kg, i.m.) and xylazine hydrochloride (1 mg/kg, i.m.), the monkeys were anesthetized with 2–3% sevoflurane. Two head holders were mounted in parallel over the skull for head fixation, and several screws were implanted into the skull as anchor. A skull portion corresponding to the frontal lobe was removed, and a plastic chamber (67 mm long × 32 mm wide × 15 mm deep) was attached onto the exposed skull. One week later, the head of each monkey who was seated in a primate chair was fixed to a stereotaxic frame attached to the chair. A glass-coated tungsten microelectrode (0.5–1.5 MΩ at 1 kHz; Alpha Omega, USA) was inserted perpendicularly into the M1, SMA, PMd, and PMv to identify their digit representations. Parameters of stimulation currents were as follows: lower than 70 μA, 200-μs duration at 333 Hz, and trains of 11 or 44 cathodal pulses. Evoked movements were carefully monitored by muscle palpation and visual inspection, thereby preparing an ICMS map of the motor-related areas.

### Golgi-Cox staining

Following ICMS mapping, the monkeys were perfused transcardially with PBS under deep anesthesia with an overdose of sodium pentobarbital (30 mg/kg, i.v.). The identified digit region in each of the frontal motor-related areas was rapidly dissected out and processed for Golgi-Cox staining (i.e., Golgi impregnation) according to the manufacturer’s protocol (FD Rapid GolgiStain Kit, FD NeuroTechnologies, Baltimore, MD, USA). In brief, blocks containing the motor-related areas were placed in a mixture of solutions A and B (1:1) for two weeks at room temperature in the dark, and the mixed solution was replaced after 24 h. The blocks were then immersed in solution C for cryoprotection for three days at 4 °C in the dark, and the solution was replaced after 24 h. Subsequently, each block was sectioned coronally at 200-μm thickness on a vibratome (Neo-LinearSlicer MT, Dosaka EM, Japan). The sections were mounted onto gelatin-coated glass slides and reacted with a mixture of solutions D and E and distilled water (1:1:2) for ten min at room temperature to visualize pyramidal neurons. After several washes in distilled water, the sections were dehydrated in graded alcohols, defatted in xylene, coverslipped, and then observed under a light microscope (Axio imager Z1) with an objective lens (63 × oil, N.A 1.4, working distance 0.19 mm, ZEISS). Dendritic spine images were taken with Axio Imager Z1 at a resolution of 150 dpi and 2D-reconstructed by Neurolucida. Black and white reversal was done to emphasize the spine shape.

### Analyses of complexity and spine density of basal dendrites in Golgi-impregnated pyramidal neurons

Based on the somal size of Golgi-impregnated pyramidal neurons and the basal dendrite structure, large layer V pyramidal neurons (i.e., putative CST neurons) in each of the motor-related areas were selected for analyses of the complexity of basal dendrites and the density of dendritic spines (for complexity analysis, 20 neurons; for spine analysis, 20 neurons, two dendrites each). Morphological analyses of these pyramidal neurons were carried out as reported elsewhere^44^. A previous work was adopted in terms of the layered structure of the motor-related areas^45^, and the criteria of basal dendrites of pyramidal neurons were in accordance with prior studies^46^. In our experiments, we selected the large layer V pyramidal neurons which had at least two basal dendrite arbors with multiple branching. The basal dendrites of such large layer V pyramidal neurons were traced using Neurolucida, and all data were incorporated into Neurolucida explorer. The complexity of the basal dendrites, comprising their total length and the number of intersections, was assessed by Sholl analysis^47^ which serves to analyze the whole structure of dendritic arbors. Using this analysis, we counted the number of intersections of single basal dendrites on concentric circles which start at 30 µm away from the center of soma and gradually increase radii by 10 µm, and then measured the length of single basal dendrites per 10 µm. For the two dendrites selected from each neuron, the density of dendritic spines was analyzed as the number per 10-µm dendritic segment. The total number of dendritic spines was counted by summing up all spines on every 10-µm segment of a single dendrite. Also, the density of spines was examined on every 20- or 50-µm segment to confirm whether the spine distribution might depend on the position of the basal dendrite. Since it has been described that there are only a few spines in the close vicinity of a soma^48^, we precluded the proximal segment within 30 µm of the dendritic origin from analysis. Moreover, the dendritic spines were classified into the following five types by their shapes^29,30^: filopodia type, length ≥ 2 μm, no head or head width < 0.7 µm; thin type, length:width > 1; stubby type, length:width < 1; mushroom type, head width ≥ 0.7 μm; branched type, spine head > 1 (Fig. 6). For each type, the density of dendritic spines was analyzed as described above. Furthermore, we randomly chose four neurons (two neurons from each monkey) in each of the motor-related areas and measured the spine length and width of thin- and stubby-type spines (Fig. 8d–g). In our analysis, spines with longer than 0.2-µm length/width were collected on account of spatial resolution. We counted the number of thin-, stubby-, and mushroom-type spines in the proximal, middle, and distal segments of a single dendrite (Fig. 8h–j). For the M1, SMA, and PMv, the proximal, middle, and distal segments were 30–50, 120–140, and 210–230 µm apart from the dendritic origin, respectively. For the PMd, the proximal, middle, and distal segments were 30–50, 90–110, and 150–170 µm apart from the dendritic origin, respectively.

### Statistical analyses

All morphological data about the basal dendrites of large layer V pyramidal neurons were statistically analyzed by using R software. The somal size of retrogradely labeled CST neurons and unlabeled neurons was analyzed by using Student’s t-test. The total basal dendrites length, total intersection number, and spine density were compared among the motor-related areas by using one-way ANOVA with the Tukey–Kramer method. For Sholl analysis, the basal dendrite length and intersection number per 10-µm segment were compared among the motor-related areas by using two-way ANOVA with the Tukey–Kramer method. The spine density on 20- or 50-µm segment in each area was compared among the segments by using two-way ANOVA with the Tukey–Kramer method. Correlation analysis was performed by using Pearson correlation coefficient. All data were expressed as mean ± SEM, and the statistical significance was accepted at *p* < 0.05.
